# Online grocery purchasing in Mississippi: associations with broadband, rurality, and household characteristics

**DOI:** 10.3389/fnut.2024.1472622

**Published:** 2025-01-06

**Authors:** Will Davis, Jordan W. Jones, Elizabeth Canales, Ayoung Kim, David R. Buys

**Affiliations:** ^1^Department of Agricultural Economics, Mississippi State University, Mississippi State, MS, United States; ^2^Economic Research Service, U.S. Department of Agriculture, Kansas City, MO, United States; ^3^Department of Biochemistry, Nutrition, and Health Promotion, Mississippi State University, Mississippi State, MS, United States

**Keywords:** online grocery purchasing, consumer behavior, broadband, SNAP, food access, COVID-19 pandemic, rural, Mississippi

## Abstract

**Introduction:**

This study investigates the sociodemographic, economic, and area characteristics associated with Online Grocery Purchasing (OGP) use among adult residents of Mississippi. Understanding these factors is important in a largely rural and low-income state to address barriers and enhance accessibility.

**Methods:**

Data were collected from a 2022 online pilot survey (*n* = 398) and secondary sources. A logistic regression model was used to analyze associations between self-reported OGP use and factors including local broadband quality, sociodemographic and economic characteristics, the local food environment, and participation in government nutrition assistance programs.

**Results:**

The analysis revealed that higher education and income levels are positively associated with the likelihood of OGP use. Conversely, age and rural residence emerged as potential barriers. Although broadband disparities are widespread across Mississippi, self-reported home internet type and local internet speeds showed inconsistent associations with OGP participation across analyses.

**Discussion:**

The findings underscore the nuanced interplay of accessibility and individual-level contexts in shaping OGP behavior. This study highlights the importance of addressing both structural and individual-level barriers to improve access to online grocery services in rural and low-income areas.

## 1 Introduction

Online Grocery Purchasing (OGP), the act of purchasing groceries online for delivery to the home or pickup at a local store, has grown substantially in the U.S. over previous decades, evolving from a niche service offered by a handful of retailers to an important modality for consumer food acquisition and a major component of the overall grocery industry. In recent years, the COVID-19 pandemic led to particularly significant growth in OGP use.

The increasing shift toward digital food retail marketplaces has made understanding how consumers interact with OGP key as consumer OGP decisions, and their associated factors, may differ from those of in-person shopping. Existing original research studies and meta-analyses have examined the consumer, household, and community-level factors influencing consumer opinion toward, and adoption of, OGP, including emotional perspectives, technology acceptance, and the role of the COVID-19 pandemic [e.g., (1–4)]. Previous work has also highlighted the relationship between OGP and other food purchasing behaviors/outcomes, including the healthfulness of purchased foods, brand choice, product exploration, and price sensitivities (5–8). The existing literature, however, tends to focus on OGP use in the broader, general context, with fewer studies focusing on OGP use among specific subgroups or geographic areas. In particular, OGP adoption and accessibility likely vary for low-income and rural consumers who may face additional hurdles to OGP participation relative to other populations.

The primary goal of this study was to pilot a new survey focused on online and in-person grocery purchasing behaviors. This pilot study aims to provide evidence related to OGP use, its potential determinants, and potential barriers to its adoption in the state of Mississippi, with particular interest paid to low-income and rural consumers. Specifically, we seek to identify associations between various respondent- and area-level characteristics and the likelihood of self-reporting any OGP use among a sample of permanent adult Mississippi residents in 2022. To provide additional context for our primary findings, we also analyze reported reasons for not using OGP among respondents reporting no OGP use, the share of respondents who used OGP for the first time only after the start of the COVID-19 pandemic, and the types of foods that respondents who used OGP tended to purchase online. These contextual results help us understand potential barriers to OGP use, the rate of growth in OGP use caused by the pandemic, and what foods households buy online, a potentially important outcome for understanding associations between OGP and nutrition.

We employ data from an online pilot survey conducted in early 2022 administered to permanent adult residents of Mississippi. This survey includes detailed questions regarding sociodemographic and economic characteristics, self-reported food purchasing behaviors and attitudes, internet access and utilization, physical and mental health status, government assistance program participation, types of travel used to purchase food, and household food security. Certain questions like any OGP use were asked both for the past 12 months (at the time of survey) and for the 12 months prior to the start of the COVID-19 pandemic (before March 2020) to identify potential variation in behaviors brought about by the pandemic. Additionally, we incorporate secondary data on area characteristics including internet speed, local food retailer access, and urban/rural status both as independent variables in our primary regression and to conduct a set of sub-group analyses. With our combined dataset, we conduct analyses to statistically identify factors associated with OGP use and examine differences in associations based on respondent- and area-level characteristics.

We aim to provide a comprehensive understanding of the potential determinants of OGP, with a particular focus on low-income, rural, and Supplemental Nutrition Assistance Program (SNAP) households, in addition to the full sample of survey participants. This stratified approach allows us to identify unique patterns and challenges faced by these groups, highlighting the potential role of sociodemographic, economic, and area characteristics in shaping OGP behaviors. Another key focus of our study is identifying the association between OGP use and local area-level broadband access/quality using data from Ookla internet speed tests. Given Mississippi’s low rate of broadband access, we investigate how internet availability and quality are associated with consumers’ likelihood of any OGP use. Our pilot-study analysis serves as a basis for refining our underlying hypotheses, survey instrument, and analytical approach for future, larger-scale, studies. The insights we provide on the increasing trend of OGP use and its associated factors among low-income and rural consumers could be relevant for policymakers seeking to address existing disparities in food access and nutrition in these or other contexts. This study adds to the growing literature on OGP use among low-income and rural populations by providing new information from consumers in Mississippi. Understanding these associations in Mississippi can provide insights applicable to comparable regions facing similar challenges.

## 2 Background

OGP, the act of purchasing groceries online for delivery to the home or pickup at a local store, has existed in the U.S. for decades, but its use grew rapidly following the onset of the COVID-19 pandemic. According to Gallup, only 11% of Americans surveyed in 2019 said they ordered groceries online at least once a month, while 81% said they never did (9). By 2021, 23% of respondents ordered groceries online at least once a month, and 28% did so by 2022 (10, 11). Additionally, consumers have spent significantly more on groceries online in recent years. Online grocery sales in March 2020 were estimated at $4.0 billion compared to $1.2 billion in August 2019 (12). Annual estimated OGP sales in 2021 totaled almost $98 billion, representing roughly 13% of total grocery spending for the year, with similar levels observed in 2022 and 2023 (13, 14).

Low-income and rural consumers often face unique barriers and limitations that can influence their shopping behaviors and access to food, both for OGP and brick-and-mortar retailers. For instance, low-income and rural households have lower access to reliable internet which is necessary for OGP. In 2021, 24.8% of households reporting annual incomes less than $25 k did not have any kind of home internet subscription compared to 2.2% of households reporting incomes above $150 k (15). Mejía (15) finds similar disparities in internet access for the households of Black or Hispanic survey respondents, limited English-speaking households, households with at least one disabled member, renter-occupied households, rural households, and households with lower educational attainment. While access to broadband internet (25/3 Mbps download/upload speed) in the U.S. has increased for all groups over time, it was not available to 17.3% of rural Americans in 2019 compared to only 1.2% of those living in urban areas (16). This disparity in broadband internet access may in turn drive further disparities in food access and nutrition given the increasing importance of OGP.

Low-income and rural communities also tend to face food access challenges compared to their counterparts. Areas with higher poverty rates are more likely to be classified as “food deserts” with limited access to a suitable variety of healthy, affordable foods (17). Rural areas have fewer supermarkets and grocery stores that are more likely to stock nutritious food options. In these areas, the number of chain and independent grocery stores has been steadily declining over time, with a shift away from traditional grocery stores toward alternative formats like dollar stores (18). These changes to the local food environment could affect the demand for groceries purchased via OGP, but they could also affect OGP home delivery availability if households are located outside a retailer’s delivery area. Even if consumers have internet access, the feasibility of OGP use depends on whether online retailers can deliver to their location or if there is a local retailer offering store pickup. Unsurprisingly, grocery delivery options are concentrated in more densely populated urban areas. In 2021, about 4.5 million people, primarily in rural areas, lived in low-income, low-food access communities outside of the delivery range of four of the most prominent food delivery companies (19). The delivery availability barrier could further entrench food access disparities among rural and low-income populations.

Similarly, rural and low-income populations have greater rates of disability which could translate to higher costs for traditional in-person grocery shopping and greater demand for grocery delivery. In 2021, 14.7% of the population in rural areas had a disability compared to 12.6% of those in urban areas. Additionally, 24.9% of working-age adults with a disability had incomes below the federal poverty line compared to 9.3% of those without a disability (20, 21).

Low-income households also tend to rely more heavily on benefits from government nutrition assistance programs like SNAP and the Special Supplemental Nutrition Program for Women, Infants, and Children (WIC) to acquire groceries. Due to the nature of these benefit programs, participants may face additional purchasing constraints when using their SNAP and WIC benefits for OGP relative to cash. As of the time of this study, SNAP benefits are only redeemable at specific retailers participating in the SNAP Online Purchasing Pilot (SNAP OPP). While the number of states and retailers participating in SNAP OPP has grown significantly since the pilot began in 2019, only being able to use benefits at a limited number of retailers remains a programmatic concern (22). Furthermore, while SNAP benefits can be used to buy groceries online at SNAP OPP-authorized retailers, they cannot be used for non-food items or to pay additional costs associated with OGP like fees and tips (23). Alternatively, while WIC benefits cannot be redeemed online at the time of this study, the USDA has proposed program modernizations that would allow for online WIC purchases (24).

A small number of studies in the current literature focus on OGP use among low-income and SNAP participant households specifically. Some previous work identifies potential barriers faced by these households in pre- and post-pandemic contexts, including associated costs like delivery fees; lack of control over the specific food items selected and their quality, especially perishable foods; distrust of the OGP process; enjoying or being used to the in-person shopping experience; and limited OGP availability in food deserts and rural areas (25–30). Low-income SNAP households were more likely to purchase groceries online when delivery was free and fast, and when discounts, coupons, and sales were offered (27, 28). For example, a sample of WIC participants in Zimmer et al. (31) identified greater convenience as a key motivator for OGP use. Findings regarding the connection between OGP and the healthfulness of foods purchased are mixed (29), but an expansion in OGP access via the state-level rollout of SNAP OPP was associated with reduced food hardship among low-income households during the early pandemic (32). Fewer studies, however, focus specifically on OGP use in rural areas. Existing OGP studies that do include rural areas are largely focused on OGP access, finding that ordering and delivery services were less accessible in rural food deserts, and that healthier foods like fresh produce were more accessible via OGP in urban areas, areas with lower poverty rates, and areas with higher internet availability (19, 25, 33). A related case study of an early SNAP OPP participating store in Opelika Alabama found that early online shoppers in March and April of 2020 tended to be younger and less likely to live in rural areas (34).

The state of Mississippi is a relevant area to study the potential determinants of, and barriers to, OGP utilization among low-income and rural populations. The majority of Mississippi’s population is rural and in 2022 had the lowest *per capita* income level of any state (35). The SNAP participation rate in Mississippi was 14.1% in 2022, the 14th highest among all U.S. states in that year (36). SNAP OGP through the OPP became available in Mississippi in August 2020, and 15 food retailers—including national, local, and online-only retailers, each with one or more locations—accepted SNAP benefits online as of June 2024 (23). Like other states, WIC benefits were generally not redeemable online in Mississippi as of June 2024. Additionally, Mississippi has the lowest rate of broadband access in the U.S., with only 86.3% of the population having access to broadband in (16). Separating areas of the state by rural and urban status paints an even starker picture, with only 75.2% of rural Mississippians having access to broadband compared to 97.8% of the state’s urban population (16). These factors make Mississippi an ideal location for investigating the factors associated with OGP use among disadvantaged rural communities.

## 3 Theoretical framework

The primary goal of this study is to identify factors associated with OGP use among different sociodemographic and economic groups in Mississippi using a combination of online pilot survey data and secondary area-level data sources. We are particularly interested in the roles of household broadband internet access/quality, urban/rural status, income, and government nutrition assistance program participation status.

We hypothesize that households with home internet access are more likely to use OGP. While terrestrial home internet access is not strictly necessary for OGP participation (e.g., households could use cellular data or internet connections available outside of the home), it is likely that the lack of home internet is a significant barrier to OGP use among at least a subset of Mississippi households given the state’s low level of broadband access. Furthermore, we hypothesize that internet quality also plays a role in OGP use. Specifically, access to higher-quality connections (e.g., more stable fixed-terrestrial broadband/fiber) or higher internet speeds make it easier for households to use the internet and may subsequently increase the likelihood of OGP use.

We hypothesize that households located in rural areas are less likely to use OGP for multiple reasons. Existing research [e.g., (19)] indicates that grocery delivery availability may be lower in some rural populations compared to urban ones, which in turn would likely reduce usage. Given the longer average travel time needed for rural consumers to acquire groceries, it is also possible that they could perceive a higher risk associated with OGP (e.g., if a grocery picker makes a mistake or chooses low-quality perishable goods). Because of these longer average travel times, however, it is also possible that rural consumers specifically may benefit more from grocery delivery—where it is available—which could lead to a positive association between rural location and OGP use.

We hypothesize that households with higher income are more likely to use OGP. Buying the same basket of groceries online can cost more than brick-and-mortar grocery purchasing because of delivery or pickup fees, tips, and OGP program subscription costs. Higher-income households are better able to afford these costs. Additionally, higher-income households may place a higher opportunity cost on their time. Because OGP can save time (e.g., less time traveling to or shopping in a store), these households may have a higher willingness-to-pay for OGP services. Further, if households perceive OGP use as risky from a quality perspective, higher-income households are likely more able and more willing to take the associated financial risk. It is also possible that retailer marketing efforts may specifically target higher-income households for these reasons.

Controlling for their relationship with income, it is not clear whether government nutrition assistance program participation would be correlated with OGP use, all else equal. WIC benefits could not be redeemed online in Mississippi in 2022, but SNAP and Pandemic Electronic Benefit Transfer (P-EBT) benefits could be used to purchase groceries online from certain retailers participating in the SNAP OPP. Compared to non-SNAP households using cash to purchase groceries online, SNAP recipients faced a restricted set of available options regarding where their benefits could be used for OGP. This restriction may lower the likelihood of certain SNAP households participating in OGP, particularly those with preferences for shopping at retailers not participating in the SNAP OPP. Participation in SNAP, WIC, and P-EBT is restricted primarily to lower-income households, so it is possible these households may also be less likely to use OGP for the reasons outlined above if we are not able to fully control for the role of income. Furthermore, participation in WIC and P-EBT is generally restricted to households with children, and in the case of some WIC households, pregnant women. It is therefore possible that the presence of children could influence OGP use, e.g., if parents tend to be busy and subsequently assign a high opportunity cost on their time.

## 4 Materials and methods

### 4.1 Pilot survey data

The pilot survey used in this study was developed by the researchers to capture a wide range of variables related to sociodemographic, economic, and behavioral characteristics. The survey was designed to gather detailed data on online and in-person grocery purchasing behaviors, internet access/use, government nutrition assistance program participation, physical/mental health, and food security. Based on state population means from the 2017–2021 American Community Survey (ACS) five-year estimates, participants were sampled using a Population Proportional to Size (PPS) sampling strategy with key demographic targets of age, race/ethnicity, and gender for the final sample (37). This approach helped to ensure that the final survey sample was representative of Mississippi’s population in terms of age, gender, and race/ethnicity. To enhance geographic representation across the state, participants were also sampled to match the proportion of individuals living in each of the state’s four extension regions: Delta, Northeast, Central, and Coastal.

The pilot survey’s inclusion criteria required participants to be 18 years of age or older and permanent residents of Mississippi. Individuals under the age of 18 or non-permanent residents of Mississippi were therefore excluded from the study.^1^ Data collection occurred from mid-January through the end of February 2022, with Qualtrics Research Services administering the survey online via their internal respondent panels.^2^ Respondents were compensated for participation directly by Qualtrics Research Services using their internal compensation rates.

The data cleaning process involved removing data from participants with incomplete responses to help ensure the robustness and reliability of our analysis. After completing this step, our final analysis sample for the pilot survey included 398 complete survey responses.^3^

Compared to Mississippi population averages, our survey oversampled low-income respondents which was not a specified PPS sampling target. This oversampling is beneficial for our study, however, given its focus on rural, low-income consumers, allowing for more nuanced sub-group analyses.

The pilot survey included a wide range of questions to capture detailed information across several domains. Sociodemographic characteristics collected included age, race/ethnicity, gender, marital status, household composition, and educational attainment for the respondent and other adults in the household. Economic characteristics included household income, full-time employment status, and job loss during the COVID-19 pandemic.^4^ Most importantly for this study, the survey also included information on both online and in-person grocery purchasing behaviors, including any self-reported OGP use before and after the COVID-19 pandemic, the share of groceries purchased online that were delivered to the home and/or picked up in store, types of groceries purchased online if any OGP is reported, monthly grocery spending amount for the household, distance traveled to the nearest grocery store, percent of groceries purchased or received from different food retailers/organizations, transportation modalities for grocery shopping, and reasons for not using OGP among non-participants.^5^ Self-reported internet use and quality data from the survey included current home internet access, type of home internet, self-reported internet quality, reported issues with home internet (slow download times, issues with streaming videos, etc.) and internet use outside the home.^6^ Government assistance program information was collected for SNAP, WIC, and Pandemic Electronic Benefit Transfer (PEBT) participation during both the past 12 months and the 12 months before the start of the COVID-19 pandemic. The program participation asked both if the household participated at any point in the past 12 months and conditional on saying yes, how many months the respondent received program benefits. The survey also asked respondents about their use of SNAP and PEBT benefits for OGP and reasons for not using SNAP or PEBT benefits to buy groceries online among program participants reporting no OGP use.

Health variables included general physical and mental health measured as a Likert scale (from 1 = “poor” to 5 = “excellent”), height in feet/inches (text-box entry), bodyweight in pounds (text-box entry), and incidence of COVID-19 infection in the past 12 months. Food security was assessed using the USDA’s Household Food Security Survey Module (HFSSM). The full set of HFSSM questions (10 adult food security questions for all households with eight additional questions for households with children) was asked both for the past 12 months and for the 12-month period before the start of the pandemic (before March 2020). Lastly, respondent location was captured using self-reported county name (drop down list of all Mississippi counties) and ZIP code (text-box entry) of residence.

Finally, in addition to our primary statistical analysis discussed below, we also use our pilot survey data to provide more context for our results. The survey asked respondents that reported not shopping online for groceries to select all from a list of potential reasons why they do not use OGP. These responses provide insight into the self-reported reasons for not using OGP, helping to identify potential barriers to participation. Furthermore, for respondents that did report any OGP use during the past 12 months, the survey asked if they shopped online for groceries for the first time after the start of the COVID-19 pandemic. We use this variable to determine how many respondents in our sample began using OGP in response to the pandemic relative to the number of respondents that had been using OGP even in the pre-pandemic period. Lastly, respondents reporting any OGP use in the past 12 months were asked to select all from a list of food categories their household purchased online, e.g., cereals and their products, meat and meat products, fruits and vegetables, etc.^7^ This variable provides insights into the types of food purchased by OGP households as opposed to just the use of any OGP which is the focus of our primary analyses.

### 4.2 Area-level secondary data

To analyze the associations of various area-level characteristics and OGP use, we merged our pilot survey data with data from secondary sources. Broadband internet availability/access is one factor that may play a significant role in consumers’ OGP decisions as it most likely depends on some form of reliable internet access. For OGP participation, consumers need some form of access to broadband or other internet types with adequate internet speed. To assess the availability and speed of broadband and mobile networks in each respondent’s ZIP code, we utilized data from “Speedtest by Ookla Global Fixed and Mobile Network Performance Maps” which measures internet speeds across 610.8 square meter tiles.^8^ Five-quarters of internet download speed data (from the first quarter of 2021 to the first quarter of 2022) were aggregated to the ZIP-code-level and merged with our individual-level survey data.

We include ZIP-code-level food retailer density measures in our analysis to capture variability in the local food environment. The number of SNAP-authorized food retailers was obtained from the U.S. Department of Agriculture, Food Nutrition Services’ Historical SNAP Retailer Locator Data (HSRLD) (38). To capture the number of SNAP authorized food retailers in each ZIP code at the time of survey, we exclude stores whose authorization ended in 2020 or began in or after 2021.^9^ While the HSRLD is expected to include a significant majority of food retailers in each respondent’s ZIP code, food retailers that are not SNAP authorized are not present. To supplement the HSRLD, we used Lightcast Business Data from DatabaseUSA^10^ to estimate the number of supermarkets and dollar store format retailers in each ZIP code. Stores classified as supercenters include Walmart Supercenters, Costco, Sam’s Club, and Target with 2017 North American Industry Classification System (NAICS) Code 452210. Supermarkets include grocery stores that are part of a chain (i.e., the data indicates they are a franchise), an independent grocery association, or had annual sales >$5MM, all of which are categorized with 2017 NAICS Code 445110 (39). Dollar stores include Dollar General, Dollar Tree, and Family Dollar Stores all classified with a 2017 NAICS Code of either 452210 or 452319.

To classify the respondents’ county of residence as urban, urban/rural mix, or rural, we use the Rural–Urban Continuum Codes (RUCC) provided by the USDA Economic Research Service (ERS).^11^ The RUCC are used to assign each U.S. county into one of nine RUCC categories based on metropolitan status by the Office of Management and Budget (OMB), core urban population, and adjacency of metropolitan areas. A RUCC of 1 represents the set of most urban counties (metropolitan) and 9 represents the least urban counties (most rural). In our study, counties with RUCC codes from 1 to 3 were defined as urban (metropolitan counties), those with codes from 4 to 6 as urban/rural mix, and those with codes from 7 to 9 as rural. As of 2024, Mississippi has 20 urban counties, 16 urban/rural mixed counties, and 46 rural counties, implying that roughly 76% of the state’s counties include at least some rural areas.

Finally, we include the total population and road length in kilometers of each ZIP code in our survey using data from the U.S. Census, which served as proxy measures of local market size and infrastructure development.^12^ Potential regional variations were accounted for using indicator variables for each of Mississippi’s four Cooperative Extension Regions (Delta, Northeast, Central, and Coastal). These regional indicators are defined by county with each county belonging to a single region.

### 4.3 Statistical methods

Our primary statistical analysis was conducted using a logistic regression model at the respondent level to identify the associations between our characteristics of interest and the likelihood of any self-reported OGP use. Specifically, the binary dependent variable of our logistic regression is an indicator variable equal to 1 if the respondent reported using any OGP in the past 12 months (at time of survey) and 0 otherwise, enabling us to investigate changes to the expected likelihood of any OGP use based on a set of respondent- and area-level independent variables. All logistic regression results are presented as changes in odds ratios.

The set of independent variables used in our model encompasses an array of sociodemographic, economic, and area characteristics. A full list of variable names, descriptions, and summary statistics for the full analysis sample are shown in Table 1. Variables include the number of adults living in the respondent’s household, respondent age (measured using multiple age range indicator variables with 35 years of age or under serving as the reference category), and race (with an indicator variable equal to 1 for Black or other race, leaving white as the reference category). Gender is measured with an indicator variable equal to 1 for male, while marital status is captured with an indicator variable equal to 1 if the respondent is married. Educational attainment is included as a binary indicator equal to 1 for respondents who have completed education beyond a high school degree (leaving high school or less as the reference category). Household income is incorporated using two self-reported household-income-range indicators, with the lowest range ($30 k or less) serving as the reference category. Respondent employment status is measured using an indicator variable equal to 1 if the respondent is employed full-time at the time of survey. We also include indicator variables for participation in nutrition assistance programs at any point during the past 12 months, specifically capturing SNAP, WIC, and P-EBT participation.

**Table 1:** Full sample summary statistics.

Variable	Description	Mean	Std. Dev.	Min	Max
Dependent variable					
OGP past 12 months	Household purchased groceries online in past 12 months.	0.42	0.49	0	1
Sociodemographic characteristics					
Number adults in HH	Number of adults in household.	2.23	1.12	1	8
Age: 35–44^a^	Age of respondent = 35–44 years.	0.21	0.41	0	1
Age: 45–54^a^	Age of respondent = 45–54 years	0.16	0.37	0	1
Age: 55+^a^	Age of respondent = 55+ years.	0.30	0.46	0	1
Black/another race	Race of respondent is Black or another race different from White.	0.34	0.47	0	1
Male	Gender of respondent = Male.	0.42	0.49	0	1
Married	Marital status of respondent = Married.	0.38	0.49	0	1
Some college or above	Highest level of education of respondent = Some college or above.	0.68	0.47	0	1
Economic characteristics					
Income $30–$70 K^b^	Total household income in 2020 = $30,001–$70,000.	0.39	0.49	0	1
Income >$70 K^b^	Total household income in 2020 > $70,000.	0.20	0.40	0	1
Employed full-time	Respondent is employed full-time.	0.44	0.50	0	1
P-EBT	Household received benefits through Pandemic-EBT (P-EBT) at any point during the past 12 months.	0.35	0.48	0	1
SNAP	Household received benefits through SNAP at any point during the past 12 months.	0.35	0.48	0	1
WIC	Household received benefits through WIC at any point during the past 12 months.	0.12	0.32	0	1
Physical and mental health					
Phys health: good to excellent^c^	Respondent reports their physical health as being good, very good, or excellent.	0.78	0.41	0	1
Ment health: good to excellent^c^	Respondent reports their mental health as being good, very good, or excellent.	0.75	0.43	0	1
Internet access					
Broadband, fiber, DSL^d^	Internet at home = Broadband, fiber, or digital subscriber line.	0.65	0.48	0	1
Satellite, mobile HS, dial-up, other^d^	Internet at home = Satellite, mobile hot spot (HS), dial-up, or other.	0.26	0.44	0	1
Area and food environment characteristics					
Rides to buy food	Household uses rides from family or friends to buy groceries.	0.30	0.46	0	1
Dist to grocery store 5–10 miles^e^	Distance traveled to the nearest grocery store = 5–10 miles.	0.37	0.48	0	1
Dist to grocery store 11–20 miles^e^	Distance traveled to the nearest grocery store = 11–20 miles.	0.17	0.38	0	1
Dist to grocery store >20 miles^e^	Distance traveled to the nearest grocery store >20 miles.	0.07	0.26	0	1
ZIP population	Total population of the respondent’s ZIP code in thousands	21.21	14.36	0.57	56.73
Urban^f^	County is classified as Urban based on USDA’s Rural–Urban Continuum Codes (RUCC).	0.49	0.50	0	1
Urban/rural mix^f^	County is classified as Urban/Rural Mix based on RUCC.	0.32	0.47	0	1
ZIP road length	Total road length of the respondent’s ZIP code area (in km).	188.97	137.30	9.54	856.81
Internet download speed	Average download speed (MBPS) in respondent’s ZIP code area.	154.24	67.79	13.57	306.95
SNAP stores per 1,000	Number of SNAP-authorized stores per 1,000 population.	1.09	0.50	0.21	5.23
Supercenter/market per 1,000	Number of supercenters and supermarkets per 1,000 population.	0.11	0.11	0	0.78
Dollar stores per 1,000	Number of dollar stores per 1,000 population.	0.20	0.13	0	1.23
Region: coastal	Respondent lives is Coastal Region of MS.	0.35	0.48	0	1
Region: central	Respondent lives is Central Region of MS.	0.29	0.45	0	1
Region: northeast	Respondent lives is Northeast Region of MS.	0.20	0.40	0	1
Number of observations		398			

OGP, Online grocery shopping; HH, Household; P-EBT, Pandemic Electronic Benefit Transfer; SNAP, Supplemental Nutrition Assistance Program; WIC, Special Supplemental Nutrition Program for Women, Infants, and Children.

^a^Reference category is <35 years.^b^Reference category is total income <$30,000.^c^Reference category is fair or poor health.^d^Reference category is no internet access at home.^e^Reference category is less than five miles.^f^Fully rural counties are the reference group.

General physical and mental health are measured using two separate indicator variables equal to 1 if the respondent reports their physical/mental health as being good, very good, or excellent, leaving respondents reporting fair or poor mental/physical health as the relevant reference categories. Self-reported home internet type is captured using separate category indicators. The first is equal to 1 if the respondent reports having some type of fixed-terrestrial-broadband (broadband, Digital Subscriber Line [DSL], and Fiber Optic); the second indicator is equal to 1 if the respondent reports having some other internet type not classified as fixed terrestrial broadband (Satellite, Mobile Hotspot, Dial-Up, or “other”^13^); and respondents reporting no home internet service as the reference category. An indicator variable equal to 1 if the respondent reports using rides from family or friends to buy groceries is included to identify associations between relying on others for transportation to buy food, alongside indicator variables for various self-reported distance ranges to the nearest grocery store, measured in miles, with less than 5 miles serving as the reference category.

Additional area-level variables of interest include total ZIP code population and county-level urban/rural status measured using RUCC, with separate indicator variables for urban and urban/rural mixed counties, leaving fully rural counties as the reference category. The total road length of the respondent’s ZIP code area, measured in kilometers, is also included. ZIP-code-level internet quality is measured using Ookla speed test data, specifically average download speeds across all Ookla speed tests performed in the respondent’s ZIP code area. The county-level local food environment is captured using separate variables for the number of SNAP-authorized retailers, supercenters and supermarkets, and dollar stores in a county, all measured per 1,000 population. Finally, we control for unobserved regional effects using indicators for Mississippi Cooperative Extension Regions, with the Northwest region serving as the reference region.

With our comprehensive set of sociodemographic, economic, and area factors, we mitigate confounding influences when estimating the associations of each variable with the likelihood of any OGP use. This approach allows us to accurately assess the associations of key characteristics, such as broadband access and SNAP participation, while accounting for variations in the broader contextual environment. Consequently, our results provide reliable insights into the factors associated with OGP utilization among adult survey respondents in Mississippi.

In addition to our primary, full sample, analysis, we also conduct several sub-group analyses to identify variations in associations across respondent and location types. First, we divide the sample by SNAP participation status, separately estimating our logistic regression model for both SNAP and non-SNAP households. We then estimate regressions for low- and high-income households with low-income households being those reporting annual household incomes of $30 k or less and high-income households reporting incomes greater than $30 k. Finally, we estimate our regression for the sub-sample of respondents living in urban counties and the sub-sample living in urban/rural mixed and rural counties based on RUCC.

## 5 Results

### 5.1 Full sample analysis

Table 2 presents results from our primary logistic regression model in which we investigate the respondent-, household-, and area-level factors associated with the likelihood of any OGP use among our full sample (*n* = 398).^14^ We report exponentiated coefficients from our logistic regression model in Table 2, in which the outcome is an indicator of any self-reported OGP use in the last 12 months. The coefficients shown represent the associations between changes in each independent variable and the odds ratio that a household engages in any OGP. Coefficient estimates greater than 1 indicate increased odds of OGP use while estimates below 1 indicate decreased odds. The independent variables include sets of respondent, household, and area-level variables related to sociodemographic, economic, and area characteristics, as well as health, home internet access, and food retailer access. Table 2 also shows 95% confidence intervals for each estimate and stars to denote level of statistical significance (^*^p < 0.10, ^**^p < 0.05, ^***^p < 0.01).

**Table 2:** Logistic regression of any online grocery purchasing in past 12 months: full sample.

	Odds ratios	95% CI
Number adults in HH	0.870	[0.674, 1.122]
Age: 35–44^a^	1.005	[0.525, 1.924]
Age: 45–54^a^	0.787	[0.372, 1.662]
Age: 55+^a^	0.214^***^	[0.102, 0.451]
Black/another race	0.729	[0.420, 1.264]
Male	1.034	[0.625, 1.712]
Married	1.585	[0.915, 2.743]
Some college or above	2.084^***^	[1.220, 3.559]
Income $30–$70 K^b^	1.708^*^	[0.960, 3.038]
Income >$70 K^b^	2.258^**^	[1.031, 4.946]
Employed full-time	1.159	[0.687, 1.954]
P-EBT	1.097	[0.521, 2.311]
SNAP	1.304	[0.618, 2.752]
WIC	2.723^**^	[1.255, 5.910]
Phys health: good to excellent^c^	1.396	[0.752, 2.593]
Ment health: good to excellent^c^	1.120	[0.593, 2.116]
Broadband, fiber, DSL^d^	1.424	[0.614, 3.304]
Satellite, mobile HS, dial-up, other^d^	3.156^**^	[1.286, 7.746]
Rides to buy food	1.340	[0.783, 2.295]
Dist to grocery store 5–10 miles^e^	0.942	[0.550, 1.612]
Dist to grocery store 11–20 miles^e^	0.997	[0.471, 2.107]
Dist to grocery store >20 miles^e^	2.043	[0.820, 5.093]
ZIP population	1.012	[0.988, 1.037]
Urban^f^	2.210^*^	[0.934, 5.231]
Urban/rural mix^f^	1.609	[0.758, 3.418]
ZIP road length	1.000	[0.998, 1.002]
Internet download speed	0.999	[0.994, 1.004]
SNAP stores per 1,000	0.829	[0.488, 1.410]
Supercenter/market per 1,000	3.459	[0.420, 28.47]
Dollar stores per 1,000	1.823	[0.265, 12.52]
Region: coastal	1.130	[0.545, 2.339]
Region: central	0.651	[0.302, 1.403]
Region: northeast	1.161	[0.501, 2.690]
Number of observations	398	
Log likelihood	−136.15	
Pseudo *R*^2^	0.218	

Coefficients reported as odds ratios. 95% confidence intervals of odds ratios in brackets created using robust standard errors. ^*^p < 0.10, ^**^p < 0.05, ^***^p < 0.01.

OGP, Online grocery shopping; HH, Household; P-EBT, Pandemic Electronic Benefit Transfer; SNAP, Supplemental Nutrition Assistance Program; WIC, Special Supplemental Nutrition Program for Women, Infants, and Children.

a Reference category is <35 years.

b Reference category is total income <$30,000.

c Reference category is fair or poor health.

d Reference category is no internet access at home.

e Reference category is less than five miles.

f Fully rural counties are the reference group.

We find statistically significant associations between certain sociodemographic and economic characteristics and the likelihood of any OGP use in the last 12 months. There appears to be an age gradient in the likelihood of OGP use in Mississippi. Compared to the youngest category of adults under age 35, the coefficient estimates in Table 2 indicate that, all else fixed, adults aged 35–44 were about as likely to use OGP, while adults aged 45–54 were about 21% less likely to use OGP, though these effects are statistically insignificant. The oldest age category of adults aged 55 and over were about 79% less likely to use OGP and this effect is statistically significant for our sample. We also find evidence that education level is associated with OGP use. Respondents with educational attainment above a high school diploma were about 108% more likely to use OGP than their counterparts with lower education levels. There also appears to be a statistically significant income gradient in Mississippi OGP usage for our sample. Compared to respondents with household incomes of $30 k or less, respondents reporting incomes in the range of $30,001 to $70 k were about 71% more likely to use OGP, and those reporting incomes above $70 k were 126% more likely to use OGP. We find no statistically significant evidence of differences in OGP usage for Mississippians who are Black or other race vs. white Mississippians, male vs. female, married vs. unmarried, employed full-time vs. not, or reporting good to excellent physical/mental health vs. fair or poor. However, while statistically insignificant, the point estimates for these characteristics suggest lower odds of OGP use associated with Black/other race, similar use for males as females, higher use for married respondents, somewhat higher use among full-time employed respondents, and somewhat higher use among those in better physical health or mental health.

We find limited evidence that government nutrition assistance program participation is associated with an increased probability of OGP use. Interestingly, we find no evidence of differences in OGP use for those receiving SNAP or P-EBT benefits—both of which were redeemable online during the study period. We estimate that SNAP participation was associated with a 30% increased likelihood of any OGP, but this association was not statistically significant. We do, however, find a statistically significant association suggesting a 172% increased likelihood of OGP use among WIC participants, despite the fact that these benefits require in-person redemption unlike SNAP and P-EBT.

We find some evidence of an association between home internet access and OGP use. Compared to respondents reporting no home internet access, those reporting that they had internet access were more likely to report OGP use, though we do not find clear evidence of a home internet quality gradient based on internet type as we initially expected. We find that respondents reporting a home internet type of broadband, fiber optic, or DSL are associated with about a 42% higher likelihood of OGP than a respondent with no home internet, though this association is not statistically significant. Internet access via other internet types of satellite, mobile hotspot, dial-up, or “other” is associated with about a 216% higher likelihood of OGP use relative to having no internet access at home. Conditional on type of reported home internet, we do not find evidence that local internet connection speed, measured as average download speed in MBPS from Ookla speed tests aggregated to the ZIP-code-level, is associated with the odds of respondent OGP use.^15^

We find little evidence of associations between OGP use and various dimensions of local food access, transportation, or other area characteristics. We find no evidence that self-reported distance to the nearest grocery store, the use of rides from friends or family to buy food, or the numbers of food retailers of various types *per capita* in the ZIP code are associated with OGP use. We also find no evidence that the total length of roads in each ZIP code, the Mississippi Cooperative Extension Region, or the population of the resident’s ZIP code are associated with OGP use. We do, however, find statistically significant evidence to suggest that a respondent’s county of residence being urban as opposed to rural is associated with a 121% higher likelihood of OGP use. Living in an urban/rural mixed county is associated with a 61% higher likelihood of any OGP use, though this association is not statistically significant.

### 5.2 Sub-group analyses

Table 3 presents logistic regression results using subsamples of our pilot survey respondents. These estimates explore potential differences in the factors associated with OGP use among key sub-populations of interest. An important caveat of our sub-group analyses is that the sub-samples are smaller, meaning that some estimates lose statistical significance potentially due to limited statistical power. The first two result columns in Table 3 present results for subsamples of respondents separated by SNAP participation status during the past 12 months (SNAP *n* = 138; non-SNAP *n* = 260). In general, we find comparable results for non-SNAP and SNAP respondents relative to the full sample, although most associations for SNAP respondents are insignificant, likely due to the reduced sample size. We find evidence that, among the non-SNAP sample, Black or other race respondents are associated with significantly lower odds of OGP use (OR = 0.425),^16^ while respondents reporting good to excellent physical health are associated with higher OGP use (OR = 3.44). Additionally, it appears that the association between WIC receipt and OGP usage observed in our full sample is driven by SNAP recipients, as the estimated coefficient for the non-SNAP sample is smaller and statistically insignificant, while the coefficient for the SNAP sample is much larger and statistically significant (OR = 5.55).

**Table 3:** Logistic regression of any online grocery purchasing in past 12 months: sub-sample analysis.

	SNAP households		Income level		Urban vs. rural counties	
	Non-SNAP	SNAP	≤$30 K	>$30 K	Urban	Rural & urban/rural mix
Number adults in HH	0.962	0.946	0.991	**0.717** ^*^	0.924	0.83
	[0.652, 1.418]	[0.620, 1.445]	[0.710, 1.382]	[0.502, 1.025]	[0.633, 1.349]	[0.581, 1.186]
Age: 35–44^a^	0.818	0.907	**0.322** ^*^	2.066	0.687	1.429
	[0.303, 2.210]	[0.270, 3.045]	[0.092, 1.127]	[0.771, 5.538]	[0.267, 1.763]	[0.538, 3.797]
Age: 45–54^a^	0.561	0.896	0.831	0.752	0.737	0.92
	[0.203, 1.547]	[0.232, 3.456]	[0.263, 2.630]	[0.255, 2.215]	[0.250, 2.173]	[0.278, 3.043]
Age: 55+^a^	**0.125** ^***^	0.363	**0.194** ^*^	**0.235** ^***^	**0.276** ^**^	**0.181** ^***^
	[0.047, 0.333]	[0.085, 1.555]	[0.033,1.154]	[0.093, 0.595]	[0.090, 0.844]	[0.061, 0.539]
Black/another race	**0.425** ^**^	1.35	1.167	**0.378** ^**^	0.773	0.562
	[0.187, 0.963]	[0.538, 3.389]	[0.385, 3.535]	[0.169, 0.849]	[0.350, 1.706]	[0.235, 1.346]
Male	0.98	1.211	1.409	0.721	0.974	0.905
	[0.504, 1.907]	[0.461, 3.184]	[0.604, 3.287]	[0.362, 1.438]	[0.458, 2.068]	[0.423, 1.935]
Married	1.672	1.494	**2.766** ^*^	1.426	**2.017** ^*^	1.239
	[0.800, 3.497]	[0.501, 4.453]	[0.853, 8.965]	[0.714, 2.850]	[0.956, 4.257]	[0.534, 2.872]
Some college or above	**2.644** ^**^	1.705	1.41	**3.088** ^***^	**2.270** ^*^	1.877
	[1.144, 6.108]	[0.687, 4.229]	[0.564, 3.522]	[1.314, 7.259]	[0.941, 5.474]	[0.846, 4.167]
Income $30–$70 K^b^	**2.519** ^**^	1.345	―	―	1.703	1.989
	[1.116, 5.688]	[0.501, 3.614]			[0.689, 4.205]	[0.822, 4.814]
Income >$70 K^b^	**2.639** ^**^	1.758	―	―	1.794	**3.486** ^*^
	[1.001, 6.957]	[0.246, 12.55]			[0.535, 6.022]	[0.989, 12.29]
Employed full-time	1.128	0.885	1.501	1.401	0.994	1.506
	[0.550, 2.313]	[0.342, 2.294]	[0.540, 4.170]	[0.695, 2.823]	[0.459, 2.151]	[0.709, 3.197]
P-EBT	0.720	0.894	1.78	0.513	1.054	1.066
	[0.214, 2.419]	[0.278, 2.879]	[0.523, 6.066]	[0.175, 1.505]	[0.352, 3.151]	[0.349, 3.260]
SNAP	―	―	2.173	1.101	1.436	1.514
			[0.627, 7.537]	[0.376, 3.223]	[0.477, 4.321]	[0.502, 4.562]
WIC	1.267	**5.554** ^***^	1.576	**17.77** ^***^	2.040	**3.679** ^**^
	[0.273, 5.873]	[1.906, 16.18]	[0.506, 4.911]	[2.872, 109.9]	[0.438, 9.497]	[1.285, 10.53]
Phys health: good to excellent^c^	**3.435** ^***^	0.699	1.399	2.11	1.43	1.518
	[1.376, 8.575]	[0.241, 2.025]	[0.480, 4.074]	[0.817, 5.451]	[0.561, 3.644]	[0.544, 4.233]
Ment health: good to excellent^c^	0.994	1.297	0.840	1.325	0.975	1.129
	[0.415, 2.378]	[0.417, 4.031]	[0.263, 2.678]	[0.509, 3.446]	[0.354, 2.686]	[0.430, 2.965]
Broadband, fiber, DSL^d^	2.48	1.194	1.553	1.413	2.146	2.076
	[0.630, 9.761]	[0.252, 5.650]	[0.443, 5.447]	[0.323, 6.186]	[0.452, 10.19]	[0.539, 7.992]
Satellite, mobile HS, dial-up, other^d^	**4.887** ^**^	3.718	**3.957** ^**^	**3.995** ^*^	**4.353** ^*^	**5.057** ^**^
	[1.237, 19.31]	[0.724, 19.08]	[1.054, 14.86]	[0.803, 19.88]	[0.824, 23.00]	[1.151, 22.23]
Rides to buy food	1.019	1.53	1.829	1.137	1.83	0.917
	[0.440, 2.358]	[0.641, 3.651]	[0.739, 4.524]	[0.504, 2.564]	[0.781, 4.290]	[0.412, 2.041]
Dist to grocery store 5–10 miles^e^	1.145	0.608	0.824	0.934	0.753	0.976
	[0.568, 2.309]	[0.213, 1.742]	[0.305, 2.227]	[0.444, 1.966]	[0.358, 1.587]	[0.403, 2.362]
Dist to grocery store 11–20 miles^e^	1.593	0.565	1.261	1.244	**3.358** ^*^	0.533
	[0.626, 4.054]	[0.120, 2.666]	[0.277, 5.731]	[0.460, 3.360]	[0.831, 13.57]	[0.185, 1.536]
Dist to grocery store >20 miles^e^	2.147	2.109	1.81	**3.331** ^*^	**4.003** ^*^	1.69
	[0.540, 8.532]	[0.452, 9.849]	[0.384, 8.533]	[0.912, 12.17]	[0.817, 19.61]	[0.552, 5.178]
ZIP population	1.026	0.972	1.015	1.020	1.006	1.026
	[0.994, 1.060]	[0.924, 1.022]	[0.964, 1.068]	[0.983, 1.059]	[0.973, 1.040]	[0.977, 1.078]
Urban^f^	**3.303** ^**^	1.833	**6.023** ^**^	1.319	―	―
	[1.004, 10.87]	[0.403, 8.339]	[1.122, 32.33]	[0.419, 4.155]		
Urban/rural mix^f^	2.122	1.771	**5.110** ^***^	0.792	―	―
	[0.761, 5.919]	[0.461, 6.801]	[1.493, 17.49]	[0.267, 2.355]		
ZIP road length	1.000	1.000	0.997	1.001	1.002	0.999
	[0.996, 1.003]	[0.996, 1.004]	[0.992, 1.003]	[0.998, 1.004]	[0.997, 1.006]	[0.996, 1.002]
Internet download speed	0.995	1.004	**0.994** ^*^	1.001	1.002	0.997
	[0.989, 1.002]	[0.996, 1.012]	[0.987, 1.001]	[0.993, 1.009]	[0.993, 1.011]	[0.991, 1.004]
SNAP stores per 1,000	0.442	0.758	0.504	0.780	0.683	1.122
	[0.164, 1.192]	[0.342, 1.681]	[0.222, 1.143]	[0.320, 1.897]	[0.334, 1.397]	[0.412, 3.054]
Supercenter/market per 1,000	**24.58** ^**^	0.989	4.299	6.665	**56.06** ^*^	1.421
	[1.005, 601.2]	[0.014, 69.99]	[0.180, 102.6]	[0.258, 172.4]	[0.615, 5110.5]	[0.127, 15.92]
Dollar stores per 1,000	**27.67** ^**^	0.335	2.593	6.019	23.41	0.968
	[1.497, 511.6]	[0.010, 11.80]	[0.100, 67.25]	[0.328, 110.6]	[0.243, 2250.9]	[0.072, 13.01]
Region: coastal	0.573	2.735	0.578	1.301	1.167	1.372
	[0.201, 1.632]	[0.818, 9.150]	[0.153, 2.178]	[0.445, 3.801]	[0.373, 3.650]	[0.408, 4.614]
Region: central	0.647	0.538	0.297	1.143	0.837	0.499
	[0.227, 1.842]	[0.134, 2.164]	[0.065, 1.352]	[0.384, 3.402]	[0.288, 2.434]	[0.104, 2.396]
Region: northeast	0.803	0.882	0.301	2.162	0.265	1.262
	[0.241, 2.674]	[0.220, 3.542]	[0.064, 1.417]	[0.672, 6.957]	[0.026, 2.744]	[0.379, 4.202]
Number of observations	260	138	162	236	194	204
Log likelihood	−136.15	−74.52	−82.41	−125.74	−110.81	−106.86
Pseudo *R*^2^	0.218	0.218	0.211	0.2288	0.1708	0.2126

Coefficients reported as odds ratios. 95% confidence intervals of odds ratios in brackets created using robust standard errors. ^*^p < 0.10, ^**^p < 0.05, ^***^p < 0.01. Bolded coefficients indicate statistical significance at the 1%, 5%, or 10% levels.

OGP, Online grocery shopping; HH, Household; P-EBT, Pandemic Electronic Benefit Transfer; SNAP, Supplemental Nutrition Assistance Program; WIC, Special Supplemental Nutrition Program for Women, Infants, and Children.

a Reference category is <35 years.

b Reference category is total income <$30,000.

c Reference category is fair or poor health.

d Reference category is no internet access at home.

e Reference category is less than five miles.

f Fully rural counties are the reference group.

Table 3 also presents the results of a similar subsample analysis in which the sample is divided into low-income (≤$30 k in annual household income, *n* = 162) and high-income (>$30 k, *n* = 236). Again, we find qualitatively similar results to the full sample, but with some notable differences. The consistent age gradient breaks down for lower-income respondents, as we find that 35–44 year olds are associated with a 68% lower likelihood of OGP use than younger adults and adults aged 45–54 years old. Being in the highest age group of 55+ is statistically significantly associated with a roughly 80% lower odds of OGP for low-income respondents. We estimate that lower-income adults are also associated with a higher likelihood to use OGP if they are married (OR = 2.77), if they live in a county that is urban/rural mixed (OR = 5.11) compared to fully rural counties, or if they live in a ZIP code with slower average internet speeds (OR = 0.994). Higher-income adults are associated with an increased likelihood of OGP use if they have fewer adults living in the household (OR = 0.72), are white (Black/other race OR = 0.38), receive WIC (OR = 17.8), or report living more than 20 miles from the nearest grocery store (OR = 3.33).

Finally, Table 3 presents the results of another subsample analysis in which the sample is divided into urban (*n* = 194) or non-urban (i.e., urban/rural mix and rural, *n* = 204) respondents. Again, we find mostly comparable results with some differences. Living in urban counties is associated with a higher likelihood of using OGP if the respondent is married (OR = 2.08). The results suggest that longer distances to the nearest grocery store are associated with a greater likelihood of urban county residents using OGP, relative to those with a grocery store within 5 miles. Respondents living in rural or urban/rural mixed counties appear to drive the relationship we find in the overall sample between WIC receipt and likelihood of OGP usage (OR = 3.68), and income also appears to be more directly associated with OGP use than in urban areas. Broadly, we find that respondents in most of these subsamples are associated with a higher likelihood of using OGP if they are educated beyond a high school degree or have satellite, mobile hotspot, dial-up, or “other” kinds of home internet access relative to no internet access; they are less likely to use OGP if they are age 55 or older.

**Figure 1:**
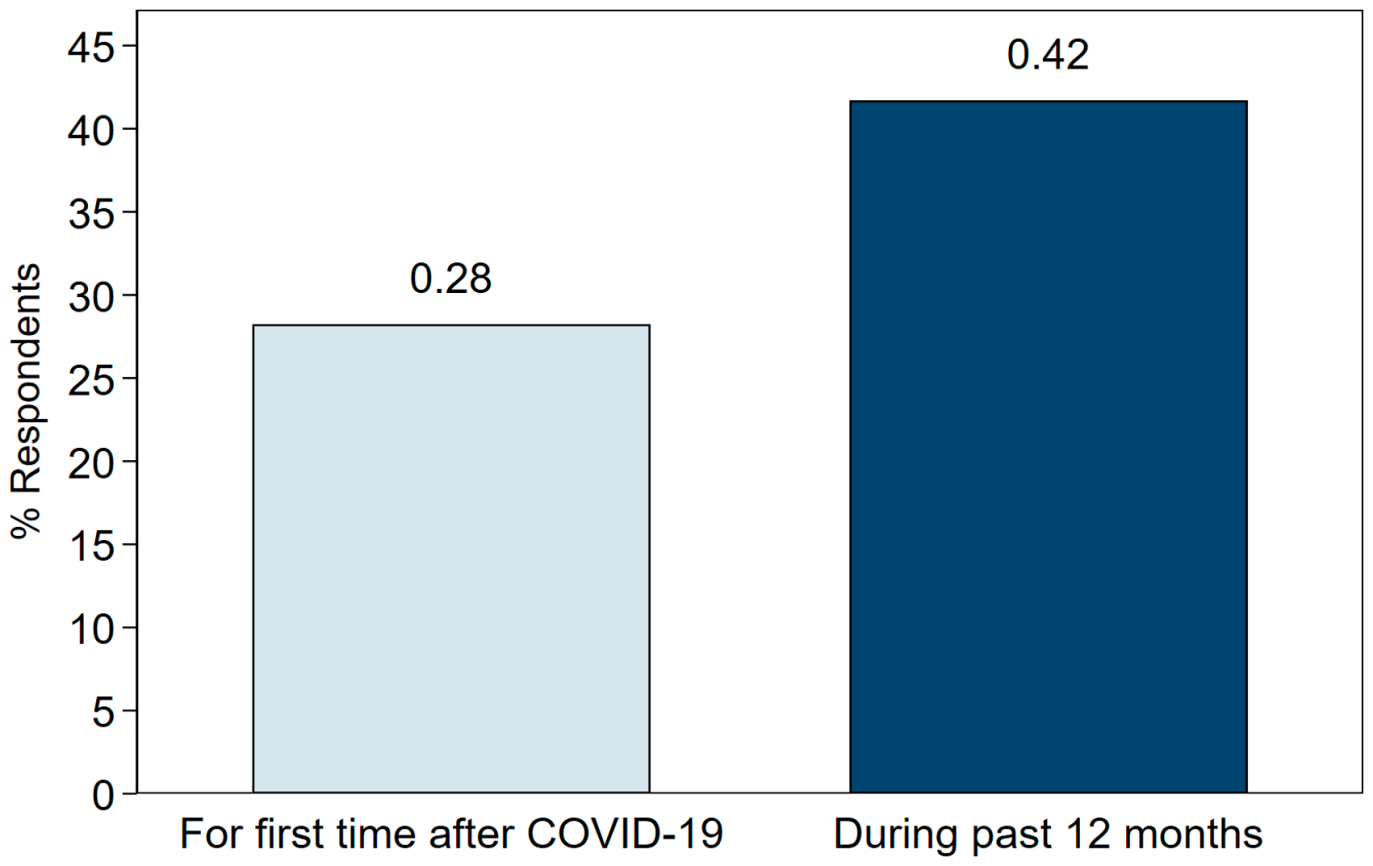
Survey respondents who reported purchasing groceries online during the past 12 months and for the first time after the COVID-19 pandemic.

Given our primary results regarding the factors associated with OGP use, we further explore household behaviors and preferences in our survey to provide additional context. As shown in Figure 1, our survey data indicates that 42% of sample respondents had purchased groceries online during the past 12 months, and 28% purchased groceries online for the first time only after the start of the COVID-19 pandemic. This implies that roughly 69% of respondents who reported purchasing groceries online in the past 12 months did so for the first time during the pandemic. This finding aligns with the increasing national trend in OGP use and highlights the COVID-19 pandemic’s role in changing how consumers purchase food and engage with e-commerce.

**Figure 2:**
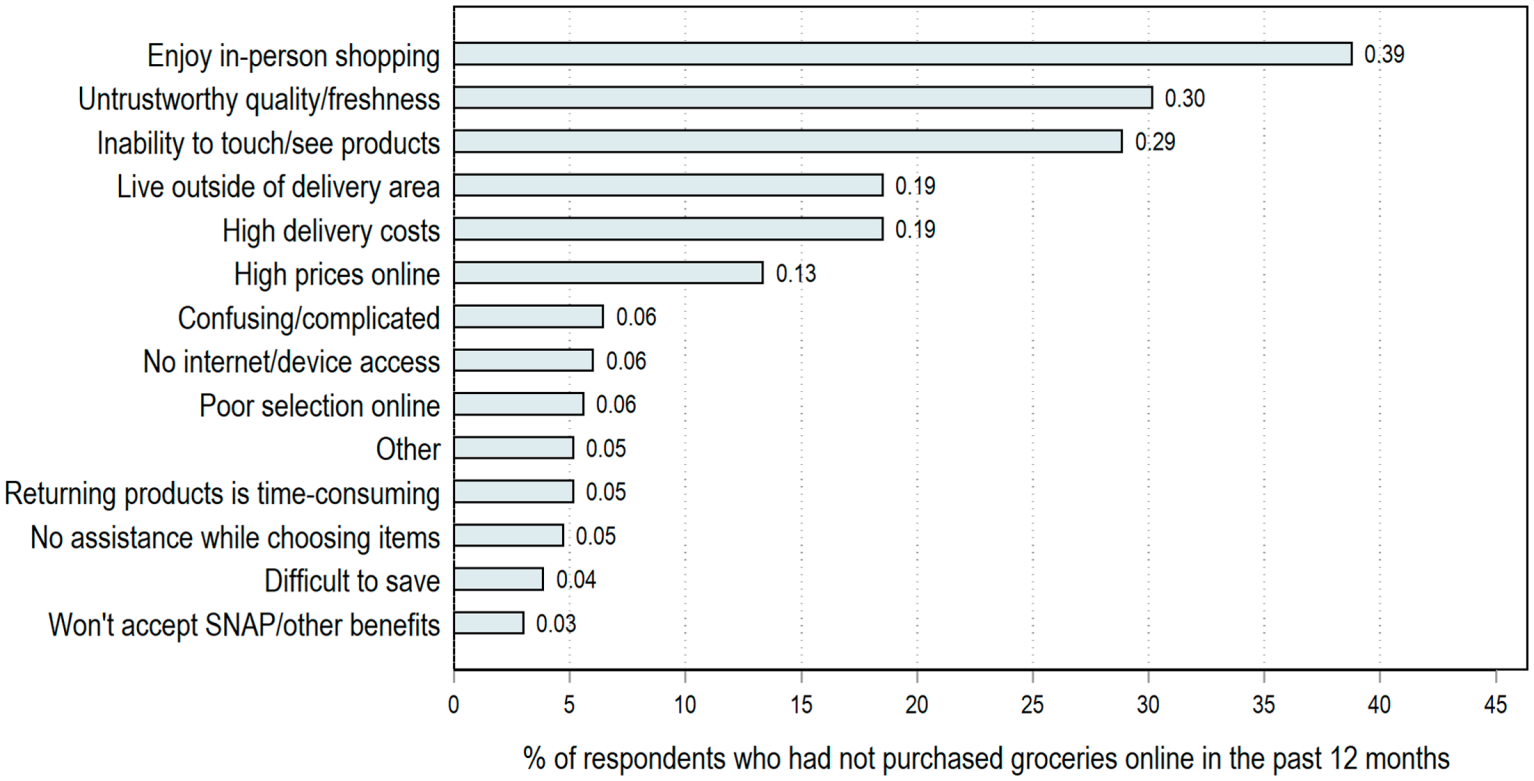
Reasons for not purchasing groceries online cited by respondents who had not purchased groceries in the past 12 months (*n* = 232). Of the total sample, 58% (232 out of 398 respondents) did not purchased groceries online in the past 12 months.

However, while there has been a significant overall increase in OGP, some consumers still rely fully on in-person grocery shopping at brick-and-mortar retailers. Figure 2 shows the responses to a question regarding reasons why respondents choose to not use OGP for those that report no-OGP use. Of these non-OGP households, 39% indicated they do not use OGP because they enjoy the in-person shopping experience, 30% cited issues related to the freshness and quality of food selected, and 29% reported the inability to see and touch the product as a reason for not used OGP. Beyond personal preferences and concerns about quality, issues related to affordability and access were also reported. For example, some respondents who had not purchased groceries online pointed to high delivery costs (19%), living outside the delivery area (19%), or high online prices (13%) as reasons for not using OGP. Surprisingly, only 6% identified lack of access to the internet or electronic devices as deterrents for purchasing groceries online. While this finding is most likely due to the pilot survey being administered online, 9% of our sample still reported not having any type of home internet. Some of the barriers to OGP are more pronounced for households located in the Mississippi Delta, a region of the state with extremely high poverty and rurality. For example, 30% of non-OGP households in the Mississippi Delta identified the lack of delivery availability where they live as a reason for not purchasing groceries online.

**Figure 3:**
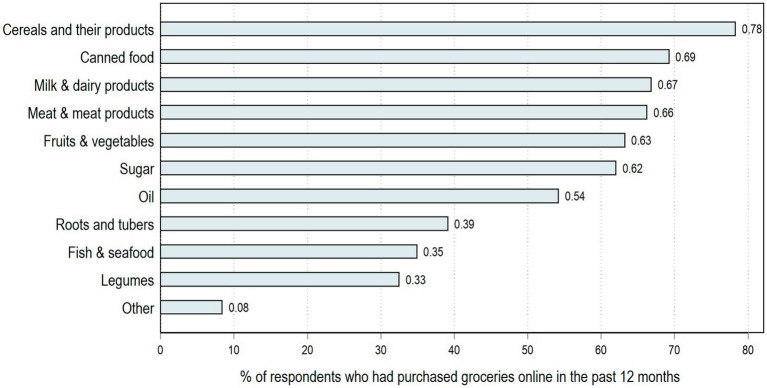
Type of groceries purchased online among survey respondents who had purchased groceries online during the past 12 months (*n* = 166). Of the total sample, only 42% (166 out of 398 respondents) purchased groceries online in the past 12 months.

Finally, we examine responses to a question asking OGP households what types of foods they purchase online to provide a sense of possible impacts on nutrition related outcomes. OGP households purchased a variety of shelf-stable and perishable products as shown in Figure 3. Around 78% of those who purchased groceries online buy cereals and their products (e.g., bread, rice, pasta, flour), while around two-thirds of OGP households purchase meats and meat products, milk and dairy products, and fruit and vegetables. These findings suggest that while the most common type of food products purchased through OGP are shelf-stable dry goods, the majority of our sample also bought fresh meat, dairy, fruit, and or vegetables, suggesting a potential avenue for improvements in diet quality among OGP households if they were not able to access these types of fresh products in their local food environment.

## 6 Discussion

Overall, our findings suggest that several individual-, household-, and area-level characteristics have statistically significant associations with the likelihood of any self-reported OGP use, including age, education, income, and living in an urban county. One possible explanation of our results is that these characteristics are associated with the potential costs and benefits of OGP. For example, younger or more educated adults may have greater familiarity with online shopping and the internet in general relative to their counterparts, making them more likely to try or continue using OGP. Younger or higher-income adults may face a greater opportunity cost of time as well which could increase their likelihood of using OGP if it is seen as more convenient. Higher-income households are also likely to be able to better afford the additional costs typically associated with OGP, like delivery fees and tips, and they may also be more willing to accept the perceived financial risks of OGP, like receiving the wrong items or poor-quality items. Living in an urban area may capture portions of the effect of greater OGP availability via delivery or pickup that is not captured by our local food retailer variables.

While we do not find evidence that SNAP participation is associated with the likelihood of OGP use, WIC participants were more likely to use OGP than nonparticipants despite the fact that, unlike SNAP and P-EBT benefits, WIC benefits were not redeemable online in Mississippi. The reasons for this association are unclear and represent a promising avenue for future research. WIC qualification indicates that a household includes a pregnant woman or mother with young children, which like other respondent characteristics, could correlate with factors like a greater opportunity cost of time when in-person shopping. It could also be that the timing of the pilot survey in relation to a national infant formula shortage is at least partially driving this relationship. Most responses to our pilot survey came from mid- to late-February 2022, which was the same month that some powdered infant formulas were recalled (40). This recall, in combination with other supply chain disruptions, made finding formula for some households difficult (40). WIC participants account for a large share of the infant formula market in the U.S., and among those reporting being affected by the formula shortage, more than 20% reported using online shopping to find formula in an existing study (40). It is possible that our findings reflect this post-formula-recall coping behavior.

Our findings suggest that home internet access is associated with the likelihood of OGP use, consistent with expectations that internet access is an essential determinant of OGP. However, we do not find evidence of a local internet quality gradient in that respondents living in ZIP code areas with higher download speeds not correlating with higher likelihood of OGP use. Additionally, our model controls for self-reported home internet access/type, implying that our local download speed variable more so represents the intensive margin of internet quality rather than the extensive margin of respondent-level home internet access. We again note that the pilot survey used in this study was conducted online, which requires that respondents have some form of internet access by default. Adults with no way of accessing the internet, either in or outside the home, could therefore not be surveyed. About 9% of the sample reported no home internet access, however, implying they still had access to the internet via internet access at work, a public place, or other means. As a result, the sample seems to use OGP more than national estimates would suggest, with 41% of our sample reporting any OGP use in the past 12 months compared to 28% nationwide in 2022 (11). It could be the case that having a connection sufficient to complete an online survey implies a connection sufficient to shop for groceries online. If this holds true, our results may best be interpreted as factors associated with OGP use for a set of Mississippians who have access to the technology needed for OGP use, but may choose not to, excluding respondents who have no ability to access OGP due to a complete lack of internet. Future iterations of this research could use phone or in-person surveys to supplement data from online surveys, allowing us to capture associations for populations with no type of internet access.

Our secondary findings also add context to understanding OGP use in areas like Mississippi with large rural and low-income populations. As seen in other areas, the COVID-19 pandemic seemed to be a catalyst for OGP growth in Mississippi as most respondents who used OGP in our sample did so for the first time following the pandemic’s onset. While many respondents in our pilot survey reported some OGP use, the majority (59%) reported not using OGP at any point in the past 12 months. Among these non-OGP respondents, the most common reasons cited for not using OGP included a preference for in-person shopping and concerns over aspects of the online shopping experience, especially concerns over the quality of items selected and the inability to see and touch products in person. These concerns could explain why the most common type of food OGP users reported purchasing online included less-perishable cereal and grain products which vary in quality less than fresh foods. Overall, these findings suggest that accessibility may not be a primary barrier to the adoption of OGP in Mississippi, although respondents in the state’s most rural and low-income area, the Delta, did report significant OGP access issues relative to the full sample.

Our findings could be used to inform targeted policy interventions through outreach or other means focused on expanding food access via OGP among diverse populations in Mississippi. For example, we find that age is negatively and statistically significantly associated with OGP in nearly all our specifications, with older adults 55 years of age and over being less likely to report using OGP at any point during the past 12 months relative to younger adults 35 and under. Additionally, we find that having a higher income is associated with a significantly higher likelihood of using OGP relative to lower-income households. This result may indicate that low-income households face greater economic barriers to OGP use relative to high-income households. While programs like the SNAP OPP are meant to expand OGP access among low-income SNAP participants, we find no evidence that SNAP participation is associated with higher OGP use in the context of Mississippi, conditional on our set of other independent variables. Our finding that higher educational attainment beyond a high school diploma is positively associated with OGP use also suggests that education may play a key role in shaping consumers’ OGP decisions, serving as a potential target for future interventions.

Addressing Mississippi’s disparity in broadband access has been a priority in recent years. For example, Mississippi’s legislature created a new office of Broadband Expansion and Accessibility of Mississippi (BEAM) in 2022 whose purpose is to expand broadband infrastructure and access in the state.^17^ However, while we find that having any type of internet access at home other than fixed-terrestrial-broadband is associated with a higher likelihood of any OGP use compared to respondents with no home internet, we do not find statistically significant associations for households with fixed-terrestrial-broadband. Our research sheds light on the potential role of home internet in OGP use, particularly in rural and low-income areas where access is limited. While we do not find that our local internet speed measure is statistically significant, the result that having some type of home internet is associated with increased OGP use still implies that economic and infrastructure policies used to decrease barriers to any internet access may play a role in expanding OGP.

We also find that WIC participation during the last 12 months is positively associated with OGP use for the full sample and the SNAP participant sub-sample, but not for non-SNAP households. This finding may indicate that respondents from households participating in both SNAP and WIC have differential behaviors related to OGP use even after controlling for respondent age, income, and gender. While WIC benefits could not be used online in Mississippi during our sample period, OGP may be a more attractive option for respondents facing greater time constraints to in-person shopping like new mothers and mothers with young children. This logic aligns with the results of Zimmer et al. (31) who found that convenience was the most often cited reason for OGP use among their sample of WIC participants. Expanding OGP access for WIC participants through changes to spending requirements, and allowing for online use similar to SNAP benefits, may further increase OGP adoption among WIC households.

Alternatively, as mentioned, we do not find a statistically significant association between SNAP participation and the likelihood of OGP use. Integrating digital solutions into government assistance programs like SNAP and WIC presents a potential method for enhancing OGP accessibility. However, while Mississippi participated in SNAP OPP during our sample period, the insignificance of SNAP participation in our primary results may indicate that SNAP households still face significant barriers to OGP adoption. Simplified online benefit redemption, technical support, and perhaps outreach campaigns to increase SNAP participants’ awareness of online redemption options through organizations like Cooperative Extension are all policy options that may bolster OGP uptake among beneficiaries. This growth could have the potential to, in turn, improve food access and nutritional outcomes among SNAP participants, especially those living in areas with limited local access to healthy food.

Lastly, fostering partnerships between retailers and local communities may help address logistical and trust barriers associated with OGP. Collaborative efforts to optimize delivery logistics, reduce fee/tip/subscription costs, and ensure product quality could enhance consumer confidence in OGP and expand adoption. As responses to our pilot survey suggest, among respondents that do not use OGP, the most commonly cited reason is enjoying the in-person shopping experience and lack of trust in the quality/freshness of products. While preference for in-person shopping reflects the tastes of the individual consumer, improvements to buying and logistical practices that increase the quality of foods purchased online may expand OGP uptake. Another commonly stated reason for not using OGP is living outside local delivery areas. Given that these respondents are also more likely to have limited access to brick-and-mortar food retailers, expanding OGP delivery areas may increase food access in underserved areas.

This pilot study does have some limitations that we acknowledge need to be addressed prior to large-scale future research. As stated, we employed an online survey to examine consumers’ OGP behaviors. The nature of an online survey inherently excludes individuals without any type of internet access at their home, work, public places, or other locations. This exclusion is not critically problematic for our study as those without any way of accessing the internet generally do not shop online. However, it may have led to the inadvertent exclusion of consumers who engage in OGP with the technical assistance of friends or family members due to a lack of internet-capable devices, access, or literacy. We are also unable to observe consumers who would like to use OGP but lack the technology to do so as the same barriers limit their ability to participate in an online survey. Furthermore, as the survey was limited to Mississippi––a state characterized by high poverty rates, rurality, and insufficient infrastructure––the results may not be representative of the average U.S. consumer. Nevertheless, the findings provide insights into the online grocery shopping trends and behaviors of households in the state.

This pilot study represents one state during the COVID-19 pandemic, which has led to significant changes in consumer behaviors including a dramatic increase in OGP use. As our results indicate, 69% of respondents in our sample reporting any OGP stated that they did so for the first time after the start of the pandemic. While national estimates suggest that the increase in OGP use has continued after the end of the pandemic, the associations in our study may prove different than estimates from studies in other states or in a post-pandemic context. Future studies in other settings could help clarify the relationships addressed in this study and whether the observed changes in OGP behaviors persisted or reverted to pre-pandemic patterns. Such research would help determine if observed behavioral changes are temporary, or indicative of long-term shifts.

## 7 Conclusion

Our study uses a combination of data from a pilot survey and secondary sources to identify factors associated with the likelihood of any self-reported OGP use in Mississippi, highlighting several relationships of note. First, sociodemographic and economic factors such as higher educational attainment and income are positively and statistically significantly associated with OGP participation, suggesting that households with lower education and income levels may face greater barriers to OGP use. We also find that age may play a role in the likelihood of OGP use with older adults being less likely to use OGP in our sample. This finding may reflect the role of a “digital divide” across the life course. Surprisingly, while we find that respondents who reported having some type of home internet other than fixed-terrestrial-broadband are more likely to use OGP than households without internet, our results do not suggest that ZIP-code-level internet download speed measured using Ookla speed test data is associated with OGP use. Future research could help to better understand the relationship between an area’s internet infrastructure and consumer behavior related to OGP.

In addition to our primary analyses, we also estimate associations between OGP use and our factors of interest for various sub-samples, including SNAP and non-SNAP households, households with low and high income, and households in urban and non-urban counties. Our results suggest that there may be unique dynamics across these groups. For example, we find that among SNAP participants, a group of specific interest in our study, the association between WIC participation and OGP use is positive and statistically significant despite in-person WIC redemption requirements. To supplement our statistical results, we also examine responses to questions regarding reasons for not using OGP, if OGP was used for the first time after the start of the COVID-19 pandemic, and the types of food purchased online by OGP households. We find that enjoying the in-person shopping experience is the most commonly reported reason for not using OGP followed by untrustworthy quality/freshness and the inability to see/touch products. Of all OGP households, 69% reported using OGP for the first time only after the start of the pandemic, and these households purchased a mix of shelf-stable and fresh foods online.

Furthermore, data from this study may inform health education efforts through organizations like Cooperative Extension, which attempt to provide interventions tailored to the needs of local communities. For example, the positive association between educational attainment and OGP found in our study may be used by Extension program planning specialists seeking to inform local populations of the potential health/nutritional impacts of OGP.

This pilot study will inform future research in several ways. First, we recognize that the pilot survey lacked questions related to certain characteristics, including respondent disability status; participation in specific OGP programs (e.g., Instacart, Walmart+, and Door Dash); and the presence of fees/tips associated with OGP for participating households. Future work is also needed to identify the cause of our high non-response rate to the question asking about number of children living in the household, a characteristic that may be driving some of our other results (e.g., the high importance of WIC participation). Additionally, as previously mentioned, solely using an online survey format for this research most likely excludes populations who do not use the internet at all by default given the need for internet access. While we did identify some respondents reporting no home internet access in our online pilot survey, future iterations of this research could use a mix of survey formats, including online, phone, and in-person surveys to capture data from various populations of interest.

Looking forward, addressing disparities in broadband infrastructure and tailoring interventions to meet the needs of diverse populations could promote more widespread adoption of OGP in Mississippi and similar states. Future studies could look beyond Mississippi to identify national trends in OGP use and the longevity of shifts in consumer behavior observed during the COVID-19 pandemic. By identifying associations between self-reported OGP use and respondent/area-level characteristics, our findings represent initial insights for policymakers, food retailers, and researchers concerned with expanding online food access through growing digital platforms in Mississippi.

## Data Availability

The datasets presented in this article are not readily available because of concerns regarding confidentiality and risk of identification, the respondent-level pilot survey data generated and analyzed in this study cannot be shared publicly. Secondary county and ZIP-code level data analyzed in this study are available upon request. Requests to access the datasets should be directed to Will Davis, gwd53@msstate.edu.

## Footnotes

^1^Upon starting the survey, respondents were taken to an Informed Consent page outlining the purpose of the study, the age and residence status requirements, and other related information (e.g., IRB approval number, procedures, the list of researchers, etc.). At the bottom of this page, respondents were asked to select from the options of “I consent to participating in this survey” and “I do not consent to participating in this survey.” Respondents selecting the second option were not asked the remaining questions, ending the survey. Consenting respondents were then asked two additional questions to verify (1) They are at least 18 years old or older, and (2) They are a permanent resident of Mississippi. Answering “no” to either of those questions immediately ended the survey.

^2^Given the potential role of internet access in consumers’ OGP decisions, solely collecting online data may prevent respondents without any form of internet access from participating in the survey. An online format was chosen for the pilot survey, however, to maximize potential sample size given financial constraints. We do, however, find that a number of respondents in our sample reported having no type of home internet. While most online survey respondents are expected to have home internet, it is entirely possible that some respondents rely on internet access outside the home, e.g., at a library, public café, etc. Regardless, only using an online survey format is a limitation of this pilot study. Future data collection building on this pilot will include offline survey data collection formats as well, e.g., phone or in-person survey data collection.

^3^One of the questions asked in the survey was the number of children living in the respondent’s household. Potentially due to the sensitive nature of the question, a significant number of respondents did not answer this question. Specifically, the analysis sample size decreases from 398 respondents if number of children is not included to 326 respondents with the number of children variable. We choose to present results without number of children to maximize our usable sample size, but results are qualitatively similar with number of children included and available upon request. Additionally, since the surveys were administered by a contracted company, Qualtrics, we are not able to observe the full survey response rate.

^4^Sociodemographic and economic questions were primarily adapted from the American Community Survey (ACS).

^5^In total, the grocery purchasing block of our pilot survey included 18 questions, with some only being asked conditional on earlier responses. Our primary question regarding any OGP use was worded as “In the past 12 months, did your household ever buy groceries online?.” The wording for this question was designed to be general, with additional questions being used to determine specifics like the share of groceries purchased online for home delivery vs. in-store pickup. Questions regarding transportation modalities were adopted from the ACS and types of groceries purchased were adapted from the National Health and Nutrition Examination Survey. Questions specific to OGP were created by the researchers. While funds were not available for full instrument validation given limited pilot study resources, the survey was launched for a brief initial period to identify any potential issues with participant response patterns (e.g., reporting the use of OGP but not reporting purchases from any specific retailers). Future iterations of the instrument will be fully validated prior to large-scale data collection.

^6^Questions regarding home internet were adapted from the ACS.

^7^Food categories were adopted from the NHANES.

^8^Speedtest by Ookla Global Fixed and Mobile Network Performance: https://registry.opendata.aws/speedtest-global-performance/.

^9^While the HSRLD allows identification of SNAP retailers, it has drawbacks. One recognized issue with the data set is duplicate observations of either a store with the same name or a store with a different name listed at the same address. These duplicates can occur for multiple reasons, including lapses in a store’s SNAP authorization, data entry errors, and multiple retailers being present at the same address in different years. Since our study only uses one year of HSRLD data, many of the potential issues caused by dynamic store changes across time are removed. That said, some stores may lose SNAP authorization for a portion of a single year, causing them to show up as two or more observations in our data set. We examined our data for this issue and found very few instances of duplicate stores. Given that this event is rare and unlikely to be correlated with other characteristics, we see it as a potential source of classical measurement error. Additionally, identifying true duplicates involves assumptions which may introduce additional bias into our analyses.

^10^Lightcast Business Data from DatabaseUSA: https://kb.lightcast.io/en/articles/6957497-business-data-from-databaseusa.

^11^USDA ERS RUCC: https://www.ers.usda.gov/data-products/rural-urban-continuum-codes/.

^12^County population data come from the American Community Survey 5-year Estimates (2017–2021): https://www.census.gov/data/developers/data-sets/acs-5year.html. Road length data come from the U.S. Census Bureau 2021 TIGER/Line Shapefiles-Roads: https://www.census.gov/geographies/mapping-files/time-series/geo/tiger-line-file.html.

^13^Respondents were allowed to select an “other” category when asked what type of home internet they had rather than answering with one of the default options. Respondents selecting “other” were then provided with a text box where they could enter the name of their other home internet type. Most respondents choosing this option wrote in identifiable types of home internet and were assigned to another category of internet type. Those that did not use the write-in text box were left in the “other” category for our analysis.

^14^Given the set of interrelated independent variables used in our logistic regressions, we used a Variance Inflation Factor (VIF) analysis to check for the presence of multicollinearity. A VIF coefficient of 5 or more is the most commonly used threshold for high multicollinearity (41). All VIF coefficients for the independent variables used in our model were well below 5, with the highest VIF being 3.79. The results of this VIF analysis are available upon request.

^15^We use only download speed rather than download speed and upload speed to avoid the high levels of multicollinearity between the two variables.

^16^“OR” represents the logistic regression coefficient measured as changes to the odds ratio of any OGP use.

^17^BEAM website: https://www.beam.ms.gov.
